# Potentially high-risk medication categories and unplanned hospitalizations: a case–time–control study

**DOI:** 10.1038/srep41035

**Published:** 2017-01-23

**Authors:** Chih-Wan Lin, Yu-Wen Wen, Liang-Kung Chen, Fei-Yuan Hsiao

**Affiliations:** 1 Graduate Institute of Clinical Pharmacy, College of Medicine, National Taiwan University, Taipei, Taiwan; 2 Clinical Informatics and Medical Statistics Research Center, Chang Gung University, Taoyuan, Taiwan; 3 Aging and Health Research Center, National Yang Ming University, Taipei, Taiwan; 4 Center for Geriatrics and Gerontology, Taipei Veterans General Hospital, Taipei, Taiwan; 5 School of Pharmacy, College of Medicine, National Taiwan University, Taipei, Taiwan; 6 Department of Pharmacy, National Taiwan University Hospital, Taipei, Taiwan

## Abstract

Empirical data of medication-related hospitalization are very limited. We aimed to investigate the associations between 12 high risk medication categories (diabetic agents, diuretics, nonsteroidal anti-inflammatory drugs (NSAIDs), anticoagulants, antiplatelets, antihypertensives, antiarrhythmics, anticonvulsants, antipsychotics, antidepressants, benzodiazepine (BZD)/Z-hypnotics, and narcotics) and unplanned hospitalizations. A population-based case–time–control study was performed using Taiwan’s National Health Insurance Research Database. Patients who experienced an unplanned hospitalization (index visit) were identified as index subjects and matched to a randomly selected reference visit within users of a specific category of high-risk medication. An unplanned hospitalization was defined as a hospital admission immediately after an emergency department visit. Discordant exposures to the high-risk medication during the case period (1–14 days before the visit) and the control period (366–379 days before the visit) were examined in both index and reference visits. Antipsychotics was associated with the highest risk of unplanned hospitalizations (adjusted OR: 1.54, 95% CI [1.37–1.73]), followed by NSAIDs (1.50, [1.44–1.56]), anticonvulsants (1.34, [1.10–1.64]), diuretics (1.24, [1.15–1.33]), BZD/Z-hypnotics (1.23, [1.16–1.31]), antidepressants (1.17, [1.05–1.31]) and antiplatelets (1.16, [1.07–1.26]). NSAIDs and narcotics were associated with the highest risks of unplanned hospitalizations with a length of stay ≥10 days. These medication categories should be targeted for clinical and policy interventions.

Adverse drug events (ADEs) and their associated morbidity and mortality^1^ represent a significant burden on the healthcare system^2^. Systemic reviews have estimated that approximately 5–10% of total hospital admissions are related to an ADE^3^,^4^. For the years 2004–2005 in the US, there were more than 700,000 ADE-related emergency department visits annually, and 1 of every 6 led to subsequent hospitalizations^5^. The annual cost of ADE in the US has been estimated at more than $ 136 billion^6^. In addition, the elderly are the most vulnerable to ADE-associated unplanned hospitalization^7^. For the elderly, the percentage of hospitalization attributed to ADE is estimated at 3.4–16.6%^8^.

Since a great proportion of ADE-related hospitalizations are preventable^4^, efficient identification of ADE-related hospitalizations is therefore crucial to help highlight area which clinicians and policy-makers can put efforts in. Previous studies have adopted different approaches to identify ADE-related hospitalizations, such as analysis of spontaneous reporting data^9^, medical chart review^5^,^7^,^8^,^10^,^11^,^12^,^13^,^14^, and screening diagnostic codes from electronic medical databases^15^,^16^,^17^. However, these methods are plagued with under-reporting issues and reporting bias. In addition, some of the methods are personnel-costly and time-consuming, resulting in studies with small sample sizes that cannot be generalized^10^,^12^,^13^,^14^. Furthermore, it is difficult to interpret the results between different medication categories in existing studies, since most studies only provide descriptive data on the frequency of ADE-related hospitalizations and do not consider the medication exposure prevalence within the population^5^,^7^,^9^,^11^,^13^,^14^,^15^,^16^,^17^.

Analytical studies do overcome the above-mentioned limitations. Nevertheless, most of the existing studies focus on one specific medication (category) and cause-specific hospitalizations, such as rosiglitazone and myocardial infarction^18^. Recently, a case–time–control study conducted in Western Australia investigated the associations between high-risk medications and unplanned hospitalizations. This study provided an alternative approach to compare the hospitalization risk among different medications^19^. However, this study was limited to the elderly population and did not assess age-specific differences. Furthermore, some suspected high-risk medications indicated in previous studies were not included in that study, such as central nervous system-acting drugs^4^.

To address these limitations, we evaluated the associations between 12 high-risk medication categories and unplanned hospitalizations. Furthermore, we conducted two secondary analyses to test two hypotheses. One is to explore whether there is any specific medication category associated with severe unplanned hospitalization (i.e. long length of stay). The other one is to test whether the association between high risk medication and unplanned hospitalization varied with age.

## Results

### Characteristics of index visits (unplanned hospitalizations) and reference visits

Characteristics of index and reference visits in each high-risk medication category are summarized in Supplementary Table S1. The number of index visits (unplanned hospitalizations) included in each medication category ranged between 1,962 (anticoagulants) and 85,301 (NSAIDs). The mean age of the index subjects who encounter an index visit ranged from 57.88–71.54 years for the different medication categories, and 43.4–53.7% of them were male. The number of Charlson comorbidity index, outpatient visits, emergency visits and drugs used during the case period were higher than that during the control period in all the medication categories (all have p < 0.05).

### Associations between high-risk medication categories and unplanned hospitalizations

The case–time–control ORs revealed that exposure to antipsychotics (aOR 1.54, 95% CI 1.37–1.73) was associated with the highest risk of unplanned hospitalizations, followed by exposure to NSAIDs (aOR 1.50, 95% CI 1.44–1.56), anticonvulsants (aOR 1.34, 95% CI 1.10–1.64), diuretics (aOR 1.24, 95% CI 1.15–1.33), BZD/Z-hypnotics (aOR 1.23, 95% CI 1.16–1.31), antidepressants (aOR 1.17, 95% CI 1.05–1.31), and antiplatelets (aOR 1.16, 95% CI 1.07–1.26). The proportion of unplanned hospitalizations attributable to antipsychotics exposure was 35.0%, followed by 33.3% for NSAIDS, 25.6% for anticonvulsant, 19.1% for diuretics, 18.8% for BZD/Z-hypnotics, and 14.0% for antiplatelets, respectively (Table 1). Sensitivity analyses by varying the length of the case and control period yielded similar results (Supplementary Table S2).

### Associations between high-risk medication categories and unplanned hospitalizations with hospital stay ≥10 days

**Figure 1:**
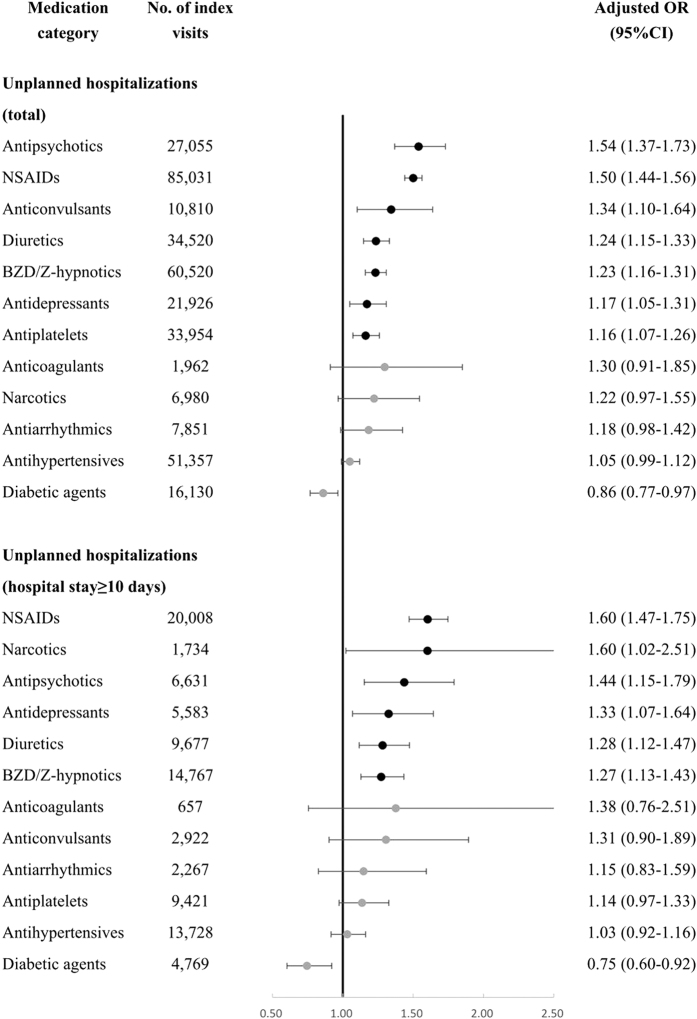
Associations between high-risk medication categories and unplanned hospitalizations/unplanned hospitalizations with hospital stay ≥10 days.

Additional analyses found that exposure to NSAIDs (aOR 1.60, 95% CI 1.47–1.75) and narcotics (aOR 1.60, 95% CI 1.02–2.51) were associated with the highest risk of unplanned hospitalizations with a length of stay ≥10 days (Fig. 1).

### Associations between high-risk medication categories and unplanned hospitalizations in aged <65 years or aged ≥65 years

**Figure 2:**
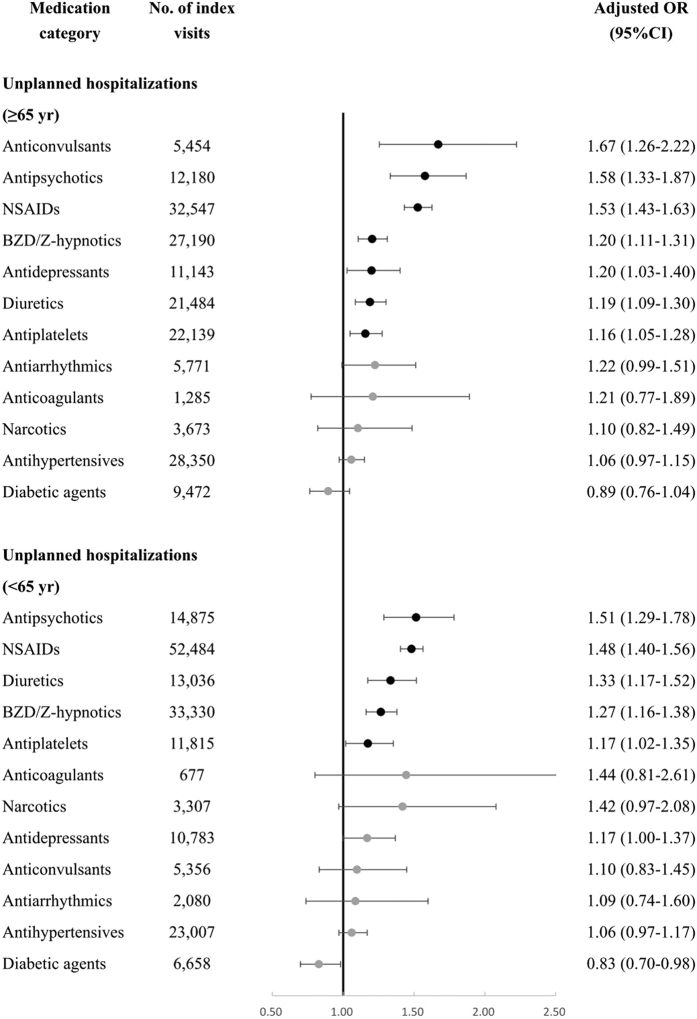
Associations between high-risk medication categories and unplanned hospitalizations in aged <65 years or aged ≥65 years.

Age-stratified analyses showed that antipsychotics, NSAIDs, diuretics, BZD/Z-hypnotics, and antiplatelets were significantly associated with unplanned hospitalizations in both aged <65 years and aged ≥65 years, but anticonvulsants and antidepressants were associated with increased risk in the elderly (aged ≥65 years) only (Fig. 2).

### Associations between individual medication classes and unplanned hospitalizations

Table 2 summarizes the subgroup analyses of the associations between individual medication classes and unplanned hospitalizations. We further identified medication classes with higher risks of unplanned hospitalization among each medication category, such as high-ceiling diuretics, non-selective and selective NSAIDs, β-blockers, classes I/III antiarrhythmics, old generation anticonvulsants, typical and atypical antipsychotics, tricyclic/tetracyclic antidepressants, long-acting and short-acting BZDs.

## Discussion

To our knowledge, our study is the first population-based study to investigate the associations between high-risk medication categories and unplanned hospitalizations in Asian adults using a case–time–control design. Among the 12 high-risk medication categories studied, we found that antipsychotics, NSAIDs, anticonvulsants, diuretics, BZD/Z-hypnotics, antidepressants, and antiplatelets were significantly associated with increased risks. In particular, NSAIDs and narcotics were associated with highest risks of unplanned hospitalizations with a length of stay ≥10 days. Our age-stratified analyses also indicated that the elderly were more vulnerable to some specific medication categories such as anticonvulsants in terms of the risk of unplanned hospitalization.

Antipsychotics and NSAIDs were associated with an increased risk of unplanned hospitalizations in the primary analysis, secondary analysis restricted to hospitalizations with long hospital stay, and age-stratified analyses. In addition, subgroup analysis suggests that more attention should be given to typical antipsychotics (aOR 1.51, 95% CI 1.33–1.71) and non-selective NSAIDs (aOR 1.50, 95% CI 1.44–1.57). Both of them were associated with a 50% increased risk of unplanned hospitalization. Typical antipsychotics are known to carry a higher risk of tardive dyskinesia and extrapyramidal symptoms than atypical antipsychotics, but existing studies have shown inconclusive results in terms of the association between the class of antipsychotics and the risk of hospitalizations^20^,^21^,^22^,^23^. The higher hospitalization risk of typical antipsychotics found in our study supports the findings reported by Al-Zakwani *et al*.^20^ and Aparasu *et al*.^21^ With regard to NSAIDs, previous studies have reported that COX-2 selective NSAIDs are associated with a decreased risk of hospitalizations for gastrointestinal adverse events^24^, and may result in fewer hospitalizations.

The risk estimates of anticoagulants, antihypertensives and antiarrhythmics derived from our study in aged ≥65 years are comparable with that from a case–time–control study in Western Australian elderly conducted by Price *et al*.^19^. However, the magnitude of the estimates from narcotics and NSAIDs are different between the two studies. Narcotics are at the highest risk in Price’s study (aOR 1.81, 95% CI 1.75–1.88) but did not show a significant association in our study (aOR 1.10, 95% CI 0.82–1.49). This discrepancy may be explained by the conservative use of narcotics in Taiwan. A previous study has reported that the consumption of narcotics in Taiwan (532 defined daily doses for statistical purposes (S-DDD) per million inhabitants per day)^25^ is much lower than that in Australia (9,031 S-DDD per million inhabitants per day)^26^. In contrast, the risk of NSAIDs was higher in our study (aOR 1.53, 95% CI 1.43–1.63) than that reported in Price’s study (aOR 1.09, 95% CI 1.06–1.12). A possible explanation is that the use of NSAIDs is captured in the NHIRD since NSAIDs are covered by the NHI in Taiwan^27^ while they are usually over-the-counter drugs in other countries.

Our study also provides the first major report of the associations between some specific high-risk medication categories and hospitalizations with long hospital stay. Descriptive data reported by McDonnell *et al*. have shown that the length of stay of ADR-related hospitalizations is different in different medication categories^28^. The extended hospitalizations may reflect not only the burden of the adverse effects but also the characteristics of users of different medications. In our study, a case–time–control design was used to eliminate bias due to the difference in characteristics between individuals, and our results indicate that the burden of ADE-related unplanned hospitalizations with long hospital stay were greatest among users of NSAIDs and narcotics.

Our age-stratified analyses extend the current understanding of high risk medications associated with unplanned hospitalization. We did this by expanding our study subjects to more than the elderly while most existing studies limited their analyses to the elderly^7^,^8^,^19^. In our study, several medication categories were significantly associated with unplanned hospitalizations in both young and elderly adults. However, we found that the elderly were more vulnerable to anticonvulsants in terms of risk of unplanned hospitalizations, but the association was not statistically significant in those <65 years old. This finding is in agreement with a prior study done by Chen *et al*. in which they report that medications requiring therapeutic drug monitoring, including anticonvulsants, are more likely to be associated with emergency visits in the elderly compared to the younger group^29^. The narrow therapeutic index of anticonvulsants as well as reduced drug clearance, polypharmacy and multimorbidity due to aging^30^ might all explain the higher hospitalization risk associated with anticonvulsant in the elderly.

The rank order of the medications with respect to their hospitalization risks in our study is inconsistent with two previous descriptive studies done in the US^5^ and Taiwan^13^. Our study shows that antipsychotics, anticonvulsants, BZD/Z-hypnotics and antidepressants are associated with higher risks of unplanned hospitalization as compared with anticoagulants, antihypertensives and diabetic agents. In contrast, previous studies have shown that anticoagulants, antihypertensives and diabetic agents caused more ADE-related emergency visits or hospitalizations than antipsychotics, anticonvulsants, BZD/Z-hypnotics and antidepressants. There may be several explanations for the differences. First, previous studies relied on detection of ADE-related hospitalizations by the physicians, so the risk of central nervous system-acting medications may be underestimated due to their wide range of non-specific adverse effects (e.g., confusion, falls, anticholinergic effects, electrolyte-imbalance, and arrhythmias)^30^. Secondly, due to the lack of the overall utilization data of each medication, descriptive studies might overestimate the risk of medications commonly used, such as cardiovascular drugs^11^,^12^. Third, descriptive studies only focus on the harm of medications^11^, whereas analytical studies take both benefit and risk into account. Therefore, antihypertensives and diabetic agents may show lower ORs of unplanned hospitalizations in our study due to their well-known preventive effects on cardiovascular events^31^,^32^.

As with any observational study based on claims databases, our study has several limitations. First, we are unable to capture variables not recorded in the NHIRD, including the severity of disease, laboratory values, lifestyle habits, and tobacco and alcohol consumptions^33^. To minimize the limitation, we used a case–time–control design to control for unchanged confounding factors and further adjusted variables changing over time in the conditional logistic models. However, unmeasured time-varying factors may still exist and can result in confounding by indication. We did conduct sensitivity analyses according to different length of case period to see if the risk of unplanned hospitalizations varied for different medications. Nevertheless, other study designs, such as case-control study, may be warranted to see if other methods draw similar conclusion as ours. Second, exposure misclassification may occur because we could not obtain the data on over-the-counter medications and herbal supplements use, as well as information about patient adherence^33^. Third, the case–time–control design is amenable to intermittent exposures and we did conduct sensitivity analyses by varying the length of case period and control period. Nevertheless, if a medication is more often used chronically, the precision of the estimates may decline due to decreased discordant pairs. Fourth, as we conducted twelve separate analyses for each potentially high risk medication category, we were unable to investigate potential synergic effects of two or more medication categories. More researches are warranted to explore this topic. Finally, odds ratios are used instead of relative risk in calculating the attributable fractions in our study, which may underestimate or overestimate the proportions of unplanned hospitalizations related to high-risk medication exposures. However, attributable fractions provide clinicians and policy makers a more straightforward picture regarding how to prioritize the risks of unplanned hospitalizations attributable to different medication categories.

## Conclusions

Antipsychotics, NSAIDs, anticonvulsants, diuretics, BZD/Z-hypnotics, antidepressants and antiplatelets were significantly associated with increased risks of unplanned hospitalizations. These medication categories should be targeted for more clinical and policy interventions.

## Methods

### Data sources

This is a population-based study using data from Taiwan’s National Health Insurance Research Database (NHIRD), a nationwide database composed of outpatient and inpatient claims for 99% of Taiwan’s population^33^. Complete data including patient demographics, information of diagnosis, prescriptions, and healthcare utilizations are well documented in the database. We used a subset of NHIRD, the Longitudinal Health Insurance Database (LHID), which contains one million beneficiaries randomly selected from the NHIRD. Claims data from 2000 to 2011 for the one million beneficiaries were extracted to compose a 12-year (2000–2011) panel of claims for analysis. Beneficiaries aged 20 years and older (adult) receiving at least one outpatient prescription during 2002 to 2011 were an additional inclusion criterion. Since the identification numbers for all of the entries in the NHIRD are encrypted to ensure privacy by the National Health Research Institute, this study was exempt from a full review by the Institutional Review Board of the National Taiwan University Hospital, and informed consent was waived (National Taiwan University Hospital Research Ethics Committee No. 201403069 W).

### Case–time–control study design

We adopted the case–time–control study design^34^,^35^, an extension of the case–crossover design^36^, to investigate the associations between high-risk medications and unplanned hospitalizations. In a case-time-control design, the identified index subjects serve both as cases and their own historical control, while background time trends in exposure are adjusted using matched reference subjects drawn from the same drug-exposure patient group as the index subjects^34^,^35^. This approach therefore, adjusts for time-invariant confounders and exposure-time trend bias resulting from changing of prescription patterns over time.

Index subjects in our study were patients who received any prescription of a specific category of high-risk medications and who experienced an unplanned hospitalization (index visit), defined as a hospital admission immediately after an emergency department visit. Individuals could be included multiple times if they had more than one index visit (i.e. unplanned hospitalization). We defined the first date of the unplanned hospitalization as the index date for each index visit. Other patients who received any prescription of the same high-risk medication category but having at least one outpatient visit (reference visit) served as the reference subjects. Each index visit was then matched to a randomly selected reference visit by age (±1 year), gender, index date (±30 days), Charlson comorbidity index^37^ (90 days prior to the index date) and number of outpatient visits (90 days prior to the index date, ±1 visit). To be eligible for the analysis, subjects also needed to have full NHI coverage for a continuous period of at least 24 months before the index date to enable the evaluation of patient history. (Supplementary Figure S1).

For both index and reference visits, we created the “case period (1–14 days before the index date)” and “control period (366–379 days before the index date)” to examine discordant exposures to the high-risk medication category between these periods. The one-year wash out period between the case period and control period was used to avoid the impact of seasonal variation on the association between exposure to high risk medications and unplanned hospitalizations. To further avoid any potential overlap of case and control periods within an individual due to repeated events, both index and reference visits needed to have no record of hospitalization 24 months before the index date. Other covariates, including Charlson comorbidity index, number of outpatient visits, number of emergency visits and number of drugs used, were retrieved 90 days prior to the case or control periods.

### Exposure to high-risk medications

Based on previous studies, our study included 12 high-risk medication categories including: diabetic agents^5^,^7^,^10^,^13^,^15^, diuretics^11^,^13^,^14^,^15^, nonsteroidal anti-inflammatory drugs (NSAIDs)^8^,^10^,^11^,^12^,^13^,^19^, anticoagulants^5^,^7^,^11^,^12^,^13^,^14^,^15^,^17^,^19^, antiplatelets^7^,^11^,^12^,^13^,^14^, antihypertensives^11^,^13^, antiarrhythmics^17^,^19^, anticonvulsants^9^, antipsychotics^8^,^9^,^12^,^14^, antidepressants^9^,^12^, benzodiazepine(BZD)/Z-hypnotics^9^,^14^, and narcotics^5^,^7^,^12^,^19^, reimbursed by Taiwan’s NHI. In addition, subgroup analyses of the association between individual medication classes and unplanned hospitalization were performed for each category except anticoagulants and antiplatelets. Exposure to high-risk medications was defined if a patient received at least one prescription of studied high-risk medication during the case or control period, and the days of supply of each prescription was considered when defining the exposure. All medications were coded according to the World Health Organization (WHO) Anatomic Therapeutic Chemical (ATC) classification system^38^, and a list of the ATC codes is presented in the Supplementary Table S3.

### Statistical analyses

For each high risk medication category, conditional logistic regression models were used to estimate the odds ratios (ORs) and 95% confidence intervals (CIs) of unplanned hospitalizations. First, crossover ORs of index visits and crossover ORs of reference visits were calculated separately. Second, the case–time–control ORs were calculated by dividing the crossover ORs of index visits by the crossover ORs of reference visits. Regression models fitted for case–time–control design included the exposure variable as well as the interaction term between the exposure and the indicator for index or reference visits, and the crude ORs were obtained from the coefficient of the interaction term^34^. Adjusted ORs were obtained from models with further adjustment for covariates including the Charlson comorbidity index, number of outpatient visits, number of emergency visits and number of drugs used.

Using adjusted ORs from the abovementioned models, we further calculated attributable fractions (AFs) to examine the proportion of unplanned hospitalizations attributable to exposure of a specific high-risk medication category using the following formula: AF (%) = [(OR − 1)/OR] × 100%^39^.

We also performed secondary analyses by restricting our index visit to unplanned hospitalizations with a length of stay ≥10 days (75th percentile of the hospital stay for unplanned hospitalizations identified in our study) to see whether some specific high-risk medication categories resulted in severe unplanned hospitalization, and by stratifying our study subjects to two age groups (<65 and ≥65 years old) to see whether the elderly were more vulnerable to some specific high-risk medication categories.

Subgroup analyses of the association between individual medication classes and unplanned hospitalization were performed for each category except anticoagulants and antiplatelets. In addition, sensitivity analyses were conducted to examine the robustness of the results by varying the length of case period and control period from 14 days to 7 and 30 days. All of the analyses were performed using SAS, version 9.2 (SAS Institute Inc., Cary, NC, USA). The p-value was two-sided, with p < 0.05 considered statistically significant.

## Additional Information

**How to cite this article:** Lin, C.-W. *et al*. Potentially high-risk medication categories and unplanned hospitalizations: a case–time–control study. *Sci. Rep.* **7**, 41035; doi: 10.1038/srep41035 (2017).

**Publisher's note:** Springer Nature remains neutral with regard to jurisdictional claims in published maps and institutional affiliations.

## Supplementary Material

Supplementary Information

## Figures and Tables

**Table 1:** Associations between high-risk medication categories and unplanned hospitalizations.

Medication category	Diabetic agents	Diuretics	NSAIDs	Anticoagulants	Antiplatelets	Antihypertensives
**Index visits**						
Exposed exclusively during the case period, no.	2,143	4,993	18,831	225	4,221	7,198
Exposed exclusively during the control period, no.	1,122	2,409	6,583	113	2,112	3,458
**Reference visits**						
Exposed exclusively during the case period, no.	1,995	3,830	13,314	162	33,05	6,061
Exposed exclusively during the control period, no.	1,013	2,559	8,893	110	21,34	3,633
**Crude odds ratio**						
Index visits crossover (95% CI)	1.91 (1.78–2.05)*	2.07 (1.97–2.18)*	2.86 (2.78–2.94)*	1.99 (1.59–2.50)*	2.00 (1.90–2.11)*	2.08 (2.00–2.17)*
Reference visits crossover (95% CI)	1.97 (1.83–2.12)*	1.50 (1.42–1.57)*	1.50 (1.46–1.54)*	1.47 (1.16–1.88)*	1.55 (1.47–1.64)*	1.67 (1.60–1.74)*
Case–time–control (95% CI)	0.97 (0.87–1.08)	1.38 (1.29–1.48)*	1.91 (1.84–1.99)*	1.35 (0.97–1.88)	1.29 (1.20–1.39)*	1.25 (1.18–1.32)*
**Adjusted odds ratio**^†^						
Index visits crossover (95% CI)	1.33 (1.22–1.44)*	1.62 (1.54–1.71)*	2.15 (2.08–2.21)*	1.63 (1.27–2.09)*	1.53 (1.44–1.62)*	1.50 (1.44–1.57)*
Reference visits crossover (95% CI)	1.54 (1.42–1.67)*	1.31 (1.24–1.38)*	1.43 (1.39–1.47)*	1.26 (0.98–1.62)	1.31 (1.24–1.39)*	1.43 (1.37–1.49)*
Case–time–control (95% CI)	0.86 (0.77–0.97)	1.24 (1.15–1.33)*	1.50 (1.44–1.56)*	1.30 (0.91–1.85)	1.16 (1.07–1.26)*	1.05 (0.99–1.12)
**Attributable fraction (AF)**						
AF(%) = [(OR − 1)/OR] × 100%^‡^ (95% CI)	−16.2% (−30.3–−3.6%)	19.1% (12.8–24.9%)	33.3% (30.6–36.0%)	23.0% (−9.8–46.0%)	14.0% (6.7–20.6%)	5.1% (−1.0–10.8%)
**Medication category**	**Antiarrhythmics**	**Anticonvulsants**	**Antipsychotics**	**Antidepressants**	**BZD/Z-hypnotics**	**Narcotics**
**Index visits**						
Exposed exclusively during the case period, no.	857	711	2,517	2,081	7,572	920
Exposed exclusively during the control period, no.	406	347	951	1,141	3,556	215
**Reference visits**						
Exposed exclusively during the case period, no.	603	480	1,363	1,627	5,758	529
Exposed exclusively during the control period, no.	379	354	946	1,201	4,083	200
**Crude odds ratio**						
Index visits crossover (95% CI)	2.11 (1.88–2.38)*	2.05 (1.80–2.33)*	2.65 (2.46–2.85)*	1.82 (1.70–1.96)*	2.13 (2.05–2.22)*	4.28 (3.69–4.96)*
Reference visits crossover (95% CI)	1.59 (1.40–1.81)*	1.36 (1.18–1.56)*	1.44 (1.33–1.57)*	1.35 (1.26–1.46)*	1.41 (1.35–1.47)*	2.65 (2.25–3.11)*
Case–time–control (95% CI)	1.33 (1.11–1.58)*	1.51 (1.25–1.82)*	1.84 (1.64–2.05)*	1.35 (1.21–1.49)*	1.51 (1.43–1.60)*	1.62 (1.30–2.02)*
**Adjusted odds ratio**^†^						
Index visits crossover (95% CI)	1.64 (1.44–1.87)*	1.57 (1.37–1.81)*	1.94 (1.79–2.11)*	1.41 (1.30–1.52)*	1.52 (1.46–1.59)*	2.47 (2.10–2.90)*
Reference visits crossover (95% CI)	1.39 (1.21–1.58)*	1.17 (1.01–1.35)*	1.26 (1.16–1.38)*	1.20 (1.11–1.30)*	1.24 (1.18–1.29)*	2.02 (1.70–2.39)*
Case–time–control (95% CI)	1.18 (0.98–1.42)	1.34 (1.10–1.64)*	1.54 (1.37–1.73)*	1.17 (1.05–1.31)*	1.23 (1.16–1.31)*	1.22 (0.97–1.55)
**Attributable fraction (AF)**						
AF(%) = [(OR − 1)/OR] × 100%^‡^ (95% CI)	15.5% (−1.6–29.8%)	25.6% (9.2–39.0%)	35.0% (26.9–42.2%)	14.7% (4.7–23.6%)	18.8% (13.8–23.6%)	18.2% (−3.4–36.3%)

*p-value < 0.05. ^†^Adjusted for Charlson comorbidity index, number of outpatient visits, number of emergency visits, number of drugs used during the case period and the control period. ^‡^Calculated using adjusted case–time–control odds ratio.

**Table 2:** Associations between individual medication classes and unplanned hospitalizations.

Medication category	Medication class	Crude OR (95% CI)	Adjusted OR (95% CI)
Diabetic agents	Oral hypoglycemic agents	0.94 (0.84–1.04)	0.84 (0.75–0.94)*
	Insulins	1.18 (0.78–1.79)	0.91 (0.60–1.39)
	Combination^†^	1.39 (1.01–1.91)*	1.09 (0.78–1.52)
Diuretics	High-ceiling diuretics	1.68 (1.46–1.93)*	1.54 (1.33–1.79)*
	Low-ceiling diuretics	1.16 (1.07–1.26)*	1.07 (0.98–1.17)
	Potassium-sparing agents	1.20 (0.84–1.72)	1.08 (0.74–1.57)
	Combination^†^	1.86 (1.60–2.16)*	1.58 (1.35–1.85)*
NSAIDs	COX-2 selective NSAIDs	1.43 (1.22–1.68)*	1.27 (1.08–1.50)*
	Non-selective NSAIDs	1.92 (1.84–1.99)*	1.50 (1.44–1.57)*
	Combination^†^	3.31 (2.60–4.22)*	2.38 (1.84–3.07)*
Antihypertensives	ACEIs/ARBs/Renin inhibitors	1.06 (0.96–1.17)	0.91 (0.82–1.02)
	CCBs	1.18 (1.07–1.29)*	1.01 (0.92–1.12)
	β-blockers	1.38 (1.24–1.52)*	1.18 (1.06–1.31)*
	α-blockers	1.35 (1.05–1.74)*	1.19 (0.91–1.55)
	Other antihypertensives	1.19 (0.89–1.58)	1.03 (0.77–1.39)
	Combination^†^	1.30 (1.20–1.41)*	1.06 (0.97–1.16)
Antiarrhythmics	Cardiac glycosides	1.12 (0.88–1.42)	1.02 (0.79–1.31)
	Antiarrhythmics, Classes I and III	1.60 (1.24–2.06)*	1.42 (1.09–1.86)*
	Combination^†^	1.17 (0.55–2.51)	0.95 (0.43–2.09)
Anticonvulsants	New generation anticonvulsants	1.19 (0.81–1.73)	1.07 (0.72–1.59)
	Old generation anticonvulsants	1.64 (1.32–2.03)*	1.45 (1.16–1.82)*
	Combination^†^	1.34 (0.58–3.14)	1.10 (0.46–2.62)
Antipsychotics	Atypical antipsychotics	1.41 (1.05–1.89)*	1.36 (1.00–1.84)*
	Typical antipsychotics	1.85 (1.64–2.08)*	1.51 (1.33–1.71)*
	Lithium	2.13 (0.59–7.75)	2.03 (0.54–7.58)
	Combination^†^	2.22 (1.32–3.73)*	1.83 (1.06–3.14)*
Antidepressants	SSRIs	1.16 (0.95–1.41)	1.01 (0.82–1.25)
	SNRIs	1.56 (0.99–2.45)	1.39 (0.87–2.23)
	SARIs	1.31 (1.04–1.65)*	1.15 (0.90–1.46)
	Tricyclics/Tetracyclics	1.38 (1.19–1.59)*	1.24 (1.07–1.45)*
	Other antidepressants	1.48 (0.98–2.24)	1.23 (0.80–1.90)
	Combination^†^	1.64 (1.24–2.16)*	1.17 (0.87–1.56)
BZD/Z-hypnotics	Z-hypnotics	1.26 (1.12–1.43)*	1.09 (0.96–1.25)
	Short-acting BZDs	1.45 (1.35–1.56)*	1.20 (1.11–1.30)*
	Long-acting BZDs	1.52 (1.36–1.69)*	1.30 (1.16–1.45)*
	Combination^†^	2.08 (1.86–2.33)*	1.45 (1.28–1.63)*
Narcotics	Weak narcotics	1.40 (1.11–1.78)*	1.10 (0.85–1.41)
	Strong narcotics	2.57 (1.29–5.09)*	1.82 (0.89–3.73)
	Combination^†^	3.27 (1.00–10.70)*	2.83 (0.80–10.03)

^*^p-value < 0.05. ^†^Combination: medications from different classes in the same category were implicated. Abbreviations: COX-2, cyclooxygenase-2; ACEI, angiotensin converting enzyme inhibitor; ARB, angiotensin receptor blocker; CCB, calcium channel blocker; SSRI, selective serotonin reuptake inhibitor; SNRI, serotonin norepinephrine reuptake inhibitor; SARI, serotonin antagonist and reuptake inhibitor.
