# Supplementary material for: Potentially high-risk medication categories and unplanned hospitalizations: a case–time–control study
Source: Sci Rep. 2017 Jan 23;7:41035. doi: 10.1038/srep41035 (PMC5253626; doi:10.1038/srep41035)
Supplement: Supplementary Information [file srep41035-s1.pdf]

Potentially high-risk medication categories and unplanned hospitalizations: a case–time–control study

Chih-Wan Lin, M.S.[1], Yu-Wen Wen, Ph.D.[2], Liang-Kung Chen, M.D., Ph.D.[3, 4], Fei-Yuan Hsiao, Ph.D.[1, 5, 6*]

[1]Graduate Institute of Clinical Pharmacy, College of Medicine, National Taiwan University, Taipei, Taiwan;

[2]Clinical Informatics and Medical Statistics Research Center, Chang Gung University, Taoyuan, Taiwan;

[3]Aging and Health Research Center, National Yang Ming University, Taipei, Taiwan;

[4]Center for Geriatrics and Gerontology, Taipei Veterans General Hospital, Taipei, Taiwan;

[5]School of Pharmacy, College of Medicine, National Taiwan University, Taipei, Taiwan;

[6]Department of Pharmacy, National Taiwan University Hospital, Taipei, Taiwan

*Corresponding author:

Fei-Yuan Hsiao, Ph.D.

Graduate Institute of Clinical Pharmacy,

College of Medicine, National Taiwan University

Room 220, 33, Linsen S. Rd, Taipei, Taiwan 10050

TEL: +886-2-33668787

FAX: +886-2-33668780

Email: fyshsiao@ntu.edu.tw

Supplementary Online Content

Supplementary Figure S1.
Study design

Supplementary Table S1.
Characteristics of index and reference visits in each high-risk medication category

Supplementary Table S2.
Sensitivity analysis: associations between high-risk medication categories and unplanned hospitalizations on different length of case and control period

Supplementary Table S3.
High-risk medications and corresponding ATC codes

Supplementary Figure S1. Study design

* Each index visit was matched to a randomly selected reference visit by index date (± 30 days), age (± 1 year), gender, Charlson comorbidity index (90 days prior to the index date) and number of outpatient visits (90 days prior to the index date, ± 1 visit).

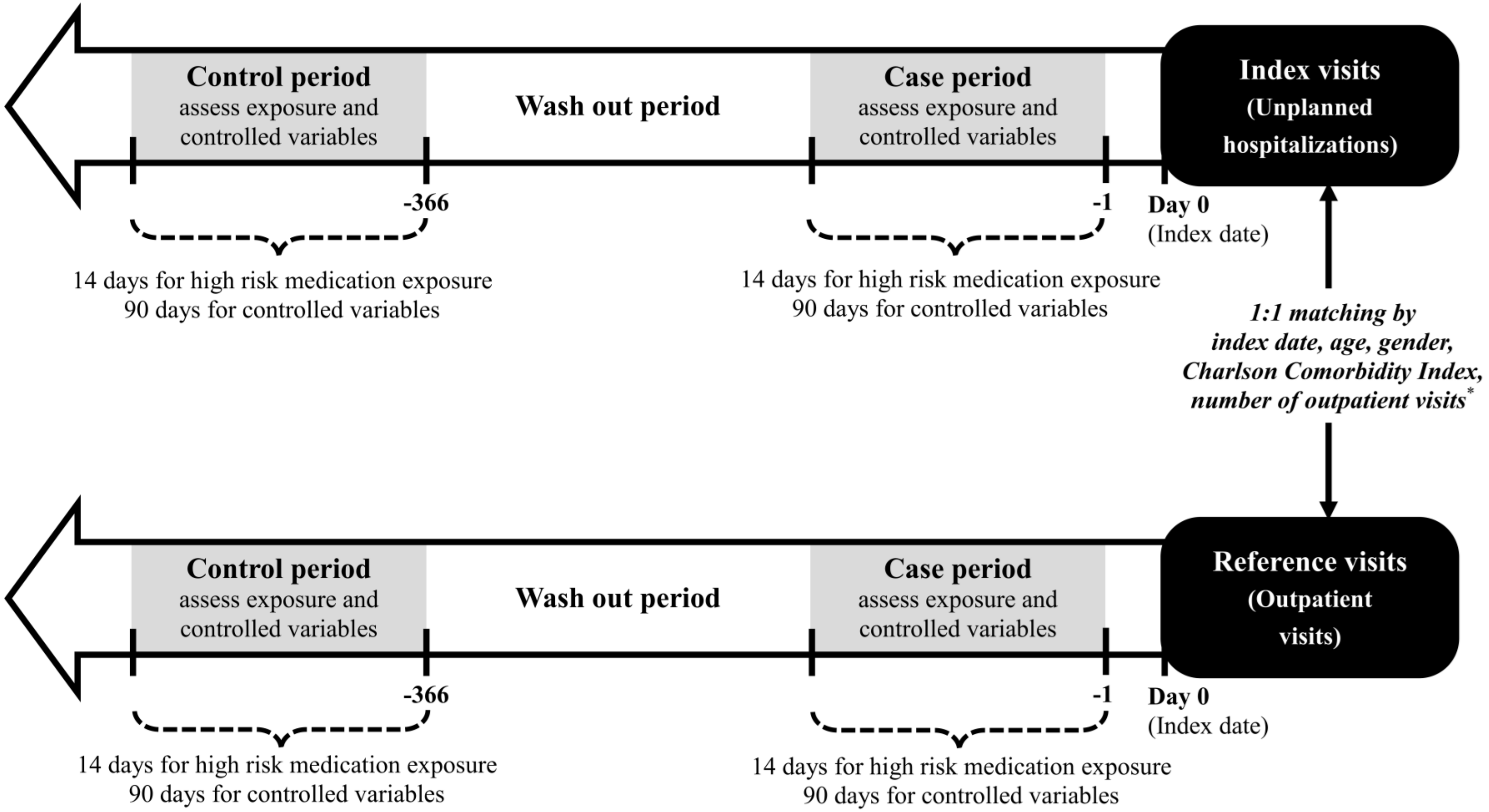

Supplementary Table S1. Characteristics of index and reference visits in each high-risk medication category

Medication category	Diabetic agents		Diuretics		NSAIDs		Anticoagulants		Antiplatelets		Antihypertensives	
	Case period	Control period	Case period	Control period	Case period	Control period	Case period	Control period	Case period	Control period	Case period	Control period
**Index visits, no.**	16,130		34,520		85,031		1,962		33,954		51,357	
Age, mean (SD)	66.90 (12.95)		68.01 (14.44)		57.88 (17.83)		69.28 (13.08)		68.96 (13.94)		65.43 (15.43)	
Male sex, no. (%)	8,079 (50.09%)		16,986 (49.21%)		45,679 (53.72%)		1,031 (52.55%)		17,497 (51.53%)		26,321 (51.25%)	
Charlson comorbidity index, mean (SD) *	1.59 (1.33)	1.37 (1.23)	1.03 (1.25)	0.85 (1.13)	1.07 (1.25)	0.90 (1.14)	0.90 (1.18)	0.75 (1.07)	0.63 (1.05)	0.51 (0.94)	1.56 (1.46)	1.39 (1.42)
No. of outpatient visits, mean (SD) *	6.60 (5.86)	5.45 (5.26)	6.56 (6.05)	5.26 (5.32)	6.61 (6.08)	5.37 (5.34)	6.03 (5.78)	4.80 (5.04)	4.71 (5.30)	3.66 (4.58)	6.90 (5.74)	5.85 (5.20)
No. of emergency visits, mean (SD) *	0.28 (0.68)	0.09 (0.39)	0.30 (0.96)	0.10 (0.42)	0.31 (0.84)	0.10 (0.42)	0.30 (0.89)	0.09 (0.42)	0.27 (0.81)	0.08 (0.37)	0.32 (0.70)	0.10 (0.37)
No. of drugs used, mean (SD) *	6.77 (4.96)	6.06 (4.68)	5.60 (4.98)	4.94 (4.67)	5.81 (4.98)	5.16 (4.68)	4.94 (4.79)	4.32 (4.47)	3.24 (4.42)	2.80 (4.06)	7.54 (5.23)	6.87 (4.84)
**Reference visits, no.**	16,130		34,520		85,031		1,962		33,954		51,357	
Age, mean (SD)	66.89 (12.93)		67.98 (14.39)		57.78 (17.78)		69.27 (13.09)		68.92 (13.89)		65.38 (15.35)	
Male sex, no. (%)	8,079 (50.09%)		16,986 (49.21%)		45,679 (53.72%)		1,031 (52.55%)		17,497 (51.53%)		26,321 (51.25%)	
Charlson comorbidity index, mean (SD) *	1.59 (1.33)	1.41 (1.21)	1.03 (1.25)	0.86 (1.13)	1.07 (1.25)	0.92 (1.15)	0.90 (1.18)	0.76 (1.07)	0.63 (1.05)	0.53 (0.95)	1.56 (1.46)	1.42 (1.42)
No. of outpatient visits, mean (SD) *	6.58 (5.78)	6.20 (5.50)	6.58 (5.93)	6.14 (5.57)	6.63 (5.96)	6.27 (5.64)	6.05 (5.65)	5.66 (5.27)	4.71 (5.18)	4.42 (4.90)	6.80 (5.59)	6.24 (5.21)
No. of emergency visits, mean (SD) *	0.10 (0.42)	0.06 (0.31)	0.10 (0.41)	0.07 (0.34)	0.10 (0.41)	0.07 (0.35)	0.09 (0.40)	0.06 (0.33)	0.08 (0.39)	0.06 (0.29)	0.16 (0.54)	0.08 (0.33)
No. of drugs used, mean (SD) *	6.55 (4.61)	6.06 (4.53)	5.57 (4.67)	5.12 (4.54)	5.72 (4.67)	5.28 (4.59)	4.91 (4.48)	4.49 (4.36)	3.25 (4.19)	2.96 (4.02)	7.35 (4.95)	6.82 (4.70)

* Paired t-tests were used to test the significance of the difference between the case period and the control period in each medication category, and all the p-values were less than 0.05.

Supplementary Table S1. Characteristics of index and reference visits in each high-risk medication category (continued)

**Medication category**	Antiarrhythmics		Anticonvulsants		Antipsychotics		Antidepressants		BZD/Z-hypnotics		Narcotics	
	Case period	Control period	Case period	Control period	Case period	Control period	Case period	Control period	Case period	Control period	Case period	Control period
**Index visits, no.**	7,851		10,810		27,055		21,926		60,520		6,980	
Age, mean (SD)	71.54 (13.94)		63.22 (16.21)		60.66 (17.69)		63.44 (16.66)		61.01 (17.72)		64.35 (15.95)	
Male sex, no. (%)	3,819 (48.64%)		5,242 (48.49%)		11,743 (43.40%)		10,047 (45.82%)		29,537 (48.81%)		3,356 (48.08%)	
Charlson comorbidity index, mean (SD) *	1.19 (1.29)	1.01 (1.20)	0.98 (1.28)	0.84 (1.17)	0.83 (1.19)	0.69 (1.08)	0.96 (1.24)	0.82 (1.14)	0.75 (1.12)	0.62 (1.01)	1.13 (1.41)	0.92 (1.27)
No. of outpatient visits, mean (SD) *	7.36 (6.54)	6.09 (5.76)	7.07 (6.71)	5.80 (5.97)	6.39 (6.21)	5.14 (5.45)	6.84 (6.35)	5.61 (5.61)	5.59 (5.67)	4.42 (4.92)	7.44 (6.80)	5.72 (5.72)
No. of emergency visits, mean (SD) *	0.35 (0.87)	0.11 (0.49)	0.37 (1.40)	0.13 (0.55)	0.33 (0.84)	0.12 (0.49)	0.35 (1.14)	0.13 (0.53)	0.30 (0.86)	0.09 (0.41)	0.40 (1.51)	0.13 (0.48)
No. of drugs used, mean (SD) *	6.88 (5.33)	6.18 (5.00)	5.60 (5.39)	4.97 (5.03)	4.58 (5.04)	4.01 (4.68)	5.61 (5.31)	4.94 (4.95)	4.07 (4.73)	3.53 (4.37)	5.79 (5.68)	4.94 (5.22)
**Reference visits, no.**	7,851		10,810		27,055		21,926		60,520		6,980	
Age, mean (SD)	71.54 (13.93)		63.21 (16.19)		60.64 (17.66)		63.42 (16.63)		60.96 (17.05)		64.34 (15.93)	
Male sex, no. (%)	3,819 (48.64%)		5,242 (48.49%)		11,743 (43.40)		10,047 (45.82%)		29,537 (48.81%)		3,356 (48.08%)	
Charlson comorbidity index, mean (SD) *	1.19 (1.29)	1.03 (1.21)	0.98 (1.28)	0.84 (1.16)	0.83 (1.19)	0.70 (1.08)	0.96 (1.24)	0.83 (1.15)	0.75 (1.12)	0.64 (1.02)	1.13 (1.41)	0.94 (1.27)
No. of outpatient visits, mean (SD) *	7.35 (6.43)	6.96 (6.12)	7.08 (6.59)	6.62 (6.16)	6.40 (6.10)	5.98 (5.74)	6.87 (6.23)	6.43 (5.85)	5.59 (5.55)	5.22 (5.21)	7.41 (6.68)	6.60 (6.01)
No. of emergency visits, mean (SD) *	0.14 (0.53)	0.08 (0.36)	0.13 (0.53)	0.08 (0.39)	0.11 (0.44)	0.07 (0.34)	0.12 (0.47)	0.08 (0.36)	0.09 (0.38)	0.06 (0.30)	0.16 (0.59)	0.09 (0.44)
No. of drugs used, mean (SD) *	6.69 (5.11)	6.26 (4.98)	5.36 (5.15)	4.96 (4.97)	4.42 (4.79)	4.08 (4.63)	5.42 (5.03)	5.03 (4.85)	4.01 (4.49)	3.65 (4.30)	5.59 (5.30)	5.03 (5.06)

* Paired t-tests were used to test the significance of the difference between the case period and the control period in each medication category, and all the p-values were less than 0.05.

Supplementary Table S2. Sensitivity analysis: associations between high-risk medication categories and unplanned hospitalizations on different length of case and control period

Medication category	Adjusted OR (95% CI)		
	Length of case period and control period		
	7 days	14 days	30 days
Diabetic agents	1.00 (0.89-1.11)	0.86 (0.77-0.97) *	0.74 (0.65-0.83) *
Diuretics	1.27 (1.17-1.36) *	1.24 (1.15-1.33) *	1.18 (1.10-1.27) *
NSAIDs	1.66 (1.59-1.74) *	1.50 (1.44-1.56) *	1.36 (1.31-1.41) *
Anticoagulants	1.26 (0.88-1.79)	1.30 (0.91-1.85)	1.07 (0.75-1.53)
Antiplatelets	1.25 (1.15-1.35) *	1.16 (1.07-1.26) *	1.10 (1.02-1.19) *
Antihypertensives	1.17 (1.10-1.24) *	1.05 (0.99-1.12)	0.93 (0.88-1.00)
Antiarrhythmics	1.29 (1.07-1.55) *	1.18 (0.98-1.42)	1.18 (0.99-1.42)
Anticonvulsants	1.27 (1.03-1.56) *	1.34 (1.10-1.64) *	1.34 (1.12-1.62) *
Antipsychotics	1.62 (1.43-1.84) *	1.54 (1.37-1.73) *	1.39 (1.26-1.55) *
Antidepressants	1.19 (1.06-1.33) *	1.17 (1.05-1.31) *	1.09 (0.98-1.21)
BZD/ Z-hypnotics	1.30 (1.23-1.39) *	1.23 (1.16-1.31) *	1.13 (1.06-1.19) *
Narcotics	1.28 (1.00-1.65) *	1.22 (0.97-1.55)	1.22 (0.99-1.50)

* p-value<0.05

Supplementary Table S3. High-risk medications and corresponding ATC codes

Medication category	Medication class	ATC codes
Diabetic agents	Insulins	A10A
	Oral hypoglycemic agents	A10B
Diuretics	High-ceiling [*]	C03C
	Low-ceiling	C03A, C03B, C02L, C07B, C07C, C07D, C09BA, C09DA, C09XA52
	Potassium-sparing agents	C03D
	Diuretics combination	C03E
NSAIDs	Non-selective	M01A(except M01AH, M01AX05 [†])
	COX-2 selective	M01AH
Anticoagulants	-	B01AA, B01AB
Antiplatelets	-	B01AC
Antihypertensives	ACEI/ARB/Renin inhibitors	C09
	CCBs	C08
	β-blockers	C07
	α-blockers	C02CA
	Other antihypertensives	C02(except C02CA)
Antiarrhythmics	Cardiac glycosides	C01A
	Classes I and III [‡]	C01B
Anticonvulsants	Old generation [§]	N03AA02, N03AB02, N03AB52, N03AF01, N03AG01
	New generation	N03AF02, N03AG04, N03AG06, N03AX09, N03AX11, N03AX12, N03AX14
Antipsychotics	Typical	N05AA01, N05AA02, N05AB02, N05AB03, N05AB04, N05AB06, N05AC02, N05AC04, N05AD01, N05AF01, N05AF02, N05AF03, N05AF05, N05AG02, N05AH01, N05AL01, N05AX09, N06CA02
	Atypical	N05AE04, N05AH02, N05AH03, N05AH04, N05AL05, N05AX08, N05AX11, N05AX12, N05AX13
	Lithium	N05AN01

**Medication category**	**Medication class**	**ATC codes**
Antidepressants	Tricyclics or Tetracyclics	N06AA, N06CA
	SSRIs	N06AB
	SNRIs	N06AX16, N06AX17, N06AX21
	SARIs	N06AX05
	Other antidepressants	N06AG02, N06AX11, N06AX12
BZD/Z-hypnotics	Long-acting BZDs	N03AE01, N05BA01, N05BA02, N05BA03, N05BA05, N05BA09, N05BA16, N05BA22, N05CD01, N05CD02, N05CD03, N05CD91, N06CA01
	Short-acting BZDs	N05BA04, N05BA06, N05BA08, N05BA12, N05BA17, N05BA56, N05BA91, N05CD04, N05CD05, N05CD06, N05CD08, N05CD09
	Z-hypnotics	N05CF
Narcotics	Strong [‖]	N02AA01, N02AB02, N02AB03
	Weak	N02AC04, N02AC54, N02AE01, N02AF02, N02AX02, N02AX52, R05DA04

NSAID= nonsteroidal anti-inflammatory drugs; COX-2=cyclooxygenase-2; ACEI= angiotensin converting enzyme inhibitor; ARB= angiotensin receptor blocker; CCB= calcium channel blocker; SSRI= selective serotonin reuptake inhibitor; SNRI= serotonin norepinephrine reuptake inhibitor; SARI= serotonin antagonist and reuptake inhibitor; BZD=benzodiazepine

[*]: loop diuretics (furosemide, bumetanide)

[†]: glucosamine

[‡]: class II (β-blockers) and class IV (CCBs) belong to antihypertensive category

[§]: phenytoin, phenobarbital, carbamazepine, valproate

[‖]: morphine, meperidine, fentanyl
